# Spinocerebellar Ataxia Type 23 (SCA23): A Rare Cause of SCA in the Americas

**DOI:** 10.1007/s12311-026-02001-6

**Published:** 2026-04-20

**Authors:** Victor Monteiro Dias Saadeh, Daniel Nassif, Luiz Felipe Vasconcellos

**Affiliations:** 1 Institute of Neurology Deolindo Couto (INDC), Federal University of Rio de Janeiro (UFRJ), Rio de Janeiro, Brazil; 2 Hospital Central da Aeronáutica (HCA), Rio de Janeiro, Brazil

**Keywords:** Movement disorders, Neurogenetics, Hereditary ataxia, Spinocerebellar ataxia, Magnetic resonance imaging

## Abstract

Spinocerebellar ataxia type 23 (SCA23) is a rare autosomal dominant hereditary ataxia caused by a pathogenic variant in the *PDYN* gene. It usually presents in adulthood, with a mean age of onset around 43 ± 15 years (reported range: 10–73 years), and progresses slowly with cerebellar symptoms. We report a case of a Brazilian 25-year-old female patient whose symptoms began at 19 years of age, characterized by progressive dysarthria, tremor, dysphagia, and gait disturbance. She had no relatives with similar symptoms. The initial genetic ataxia panel, which included the most prevalent hereditary ataxia genes, was negative. Subsequent next-generation sequencing identified a pathogenic variant in the *PDYN* gene, confirming the diagnosis of SCA23. Brain MRI demonstrated significant cerebellar atrophy. The patient was referred to a multidisciplinary rehabilitation group with emphasis on functional rehabilitation of gait and dysphagia. This case is notable for the rarity of SCA23 in the Americas, the relatively young age at onset compared with the reported mean age in the literature, while still remaining within the previously described age range, and the absence of a family history of ataxia or other neurological symptoms, despite SCA23 having an autosomal dominant inheritance pattern.

## Introduction

Spinocerebellar ataxias (SCAs) are a heterogeneous group of genetic neurodegenerative disorders characterized by progressive cerebellar ataxia. They are autosomal dominant conditions, with more than 50 subtypes identified to date [1]. Although genetic ataxias are considered rare, in specialized centers, they could represent 33% of all ataxic patients [2]. The most prevalent SCAs in Brazil, also in Latin America, are spinocerebellar ataxia type 3 (SCA3), also known as Machado–Joseph disease, followed by SCA7 and SCA2, which is consistent with the distribution patterns reported worldwide [3]. SCA23 was first described in 2004, when Verbeek et al. reported a Dutch family affected over three generations, identifying a pathogenic locus on chromosome region 20p13-12.3 through linkage analysis [4]. Later, in 2010, Bakalkin et al. identified pathogenic variants in the PDYN gene as the cause of SCA23 [5]. Also, Saigoh et al. described the first case in Japan, Liu et al. reported a case in China, Pedroso et al. in Brazil, and Fogel et al. in the United States [6–9]. Therefore, SCA23 remains a rarely reported subtype in the Americas and worldwide.

## Case Report

This is a descriptive and exploratory case study of a Brazilian 25-year-old female patient. The patient’s symptoms began at 19 years of age, with progressive dysarthria, asymmetric rest and action tremor of the upper limbs, more pronounced in the left upper limb, and handwriting difficulty. By the age of 21, she developed ataxic gait, with frequent falls requiring assistance to walk, and dysphagia.

**Fig. 1:**
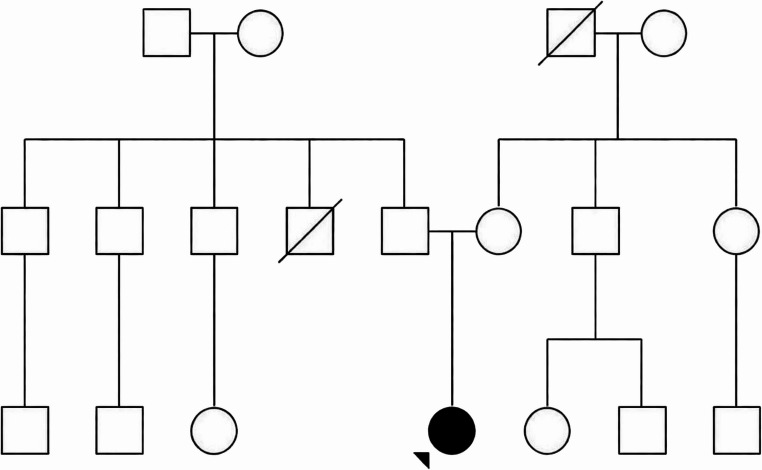
Pedigree

There was no consanguinity or family history of similar symptoms. Both parents underwent a detailed neurological examination, which was normal, with no signs of gait disturbance, dysarthria, limb ataxia, oculomotor abnormalities, or other neurological deficits. The patient has no siblings. Extended family history revealed two maternal aunts, one maternal uncle, and five paternal uncles, as well as six first-degree cousins, all reportedly neurologically asymptomatic (Fig. 1).

The exam revealed gait ataxia, dysarthria, horizontal gaze-evoked nystagmus with hypermetric saccades, hypotonia, normal strength, generalized areflexia, flexor plantar response, appendicular dysmetria in all four limbs, no sensory abnormalities with asymmetric rest and action tremor of the upper limbs.

**Fig. 2:**
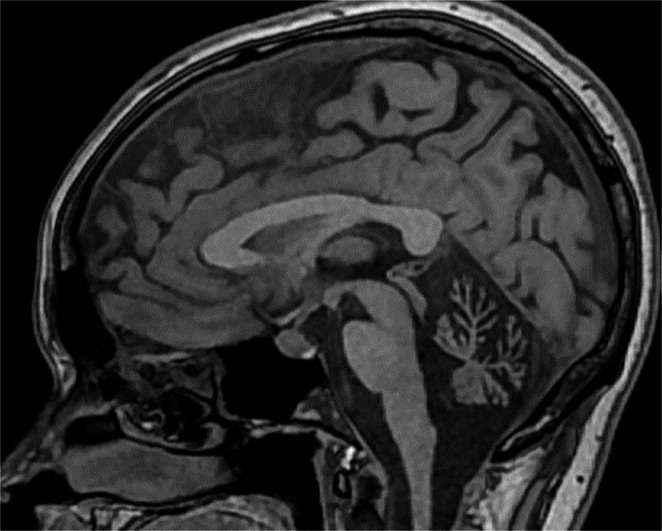
Sagittal T1-weighted image showing pronounced cerebellar atrophy, with preservation of supratentorial structures

**Fig. 3:**
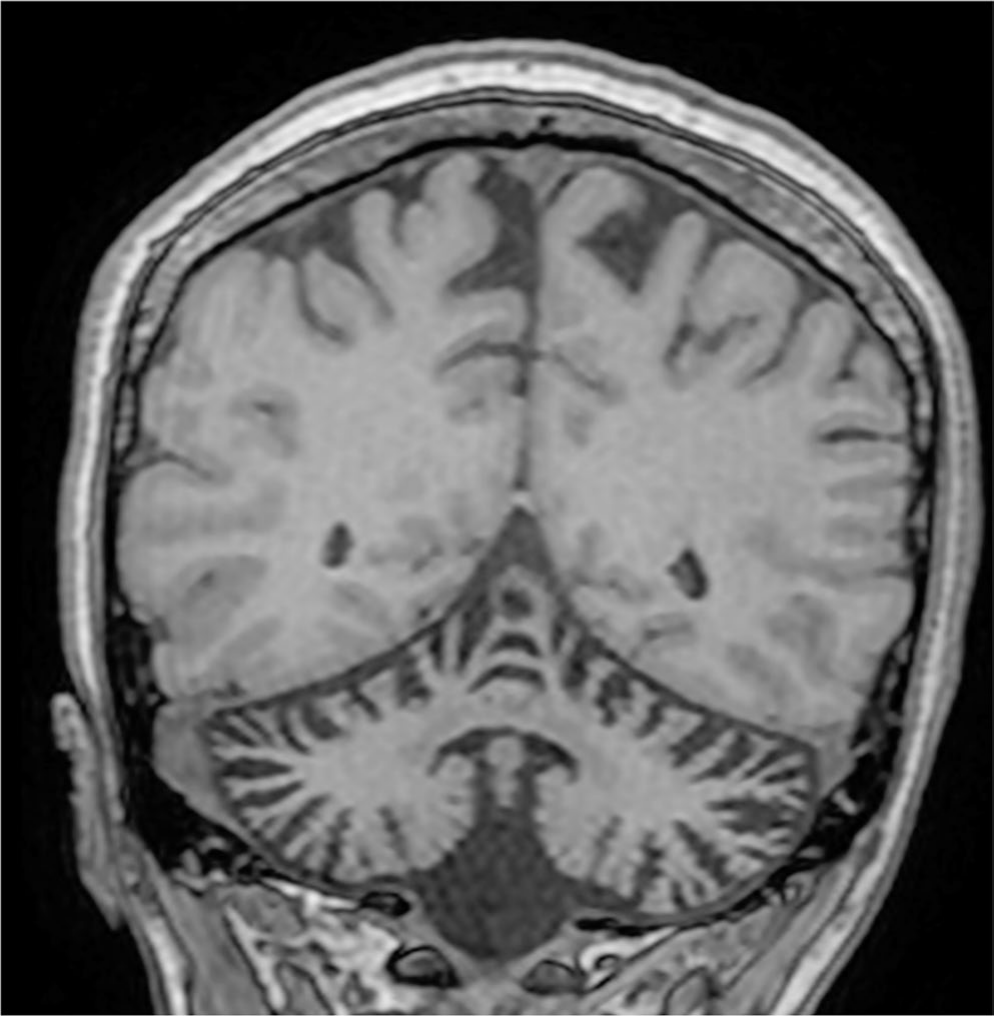
Coronal T1-weighted brain MRI demonstrates marked global cerebellar atrophy

Brain magnetic resonance imaging (MRI) revealed marked difuse cerebellar atrophy involving both cerebellar hemispheres and vermis, with no abnormalities detected in the brainstem, cerebral cortex, or corpus callosum (Figs. 2 and 3).

Acquired and potentially treatable causes of ataxia, including toxic, nutritional, and paraneoplastic etiologies, were excluded. Subsequently, genetic testing for SCA types 1, 2, 3, 6, 7, 8, and 10, as well as for Friedreich’s ataxia and fragile X syndrome, were all negative.

Genomic DNA was obtained from a buccal swab sample and analyzed using a next-generation sequencing (NGS) panel. The test included sequence analysis and deletion/duplication testing of 728 genes associated with hereditary neurological disorders.

This analysis identified a heterozygous pathogenic variant in the PDYN gene, c.414G > T (p.Arg138Ser), thereby confirming the diagnosis of SCA23. This missense variant results in the substitution of arginine, a basic and polar amino acid, with serine, a neutral and polar amino acid, at codon 138 of the prodynorphin protein. The variant is present at very low frequency in population databases (rs267606941; gnomAD ~ 0.01%) and has been previously reported in individuals with clinical features of spinocerebellar ataxia, with documented segregation with disease in affected families [5, 9]. 

The patient currently receives multidisciplinary follow-up, including physical, speech, occupational, and equine therapy.

## Discussion

The SCA23 is characterized by gait ataxia, limb ataxia, dysarthria, and hyperreflexia, with gait ataxia being the most common initial symptom. Other possible symptoms include oculomotor impairment or saccade slowing, Babinski’s sign, tremor, and polyneuropathy. However, no specific clinical findings are able to distinguish between SCA23 from other SCAs [10]. It typically presents in middle-aged adults, with a mean onset of 43 ± 15 years and a reported age range of 10–73 years. In the present case, symptom onset occurred at age 19, which is younger than the mean reported age at onset, although still within the previously described spectrum of disease presentation [5, 9, 10]. 

Reported prevalence is low: in cohort studies of ataxia patients, SCA23 accounted for 0.36–1.08% of cases. The rarity of this condition may also be related to the limited inclusion of the PDYN gene in routine genetic panels for patients who test negative for the most common SCA subtypes. ^6,7,10^

Neuroimaging, particularly MRI, can reveal abnormalities even before clinical symptoms appear, aiding early diagnosis. The most common finding in SCA23 is cerebellar atrophy, involving the cerebellar cortex, middle cerebellar peduncles, cerebral cortex, and corpus callosum [10]. In our patient, brain MRI revealed severe cerebellar atrophy as the primary radiological abnormality.

SCA23 is a progressive and incurable neurodegenerative disorder, typically autosomal dominant. There was no family history in the present case; however, in the absence of parental genetic testing, it is not possible to formally conclude that this is a *de novo* mutation. Alternative explanations, including reduced penetrance or unrecognized familial transmission, should also be considered. A similar finding was previously described by Fogel et al., who reported the only known case of SCA23 associated with a *de novo* pathogenic variant [9]. Although progression is usually slow, patients may develop significant motor disability over time [11]. This underscores the importance of multidisciplinary care — including physical, occupational, and speech therapy — alongside regular neurologic follow-up with specialists.

## Conclusion

SCA23 is a rare cause of adult-onset hereditary ataxia, typically affecting middle-aged individuals. Its true prevalence may be underestimated, given the limited availability of expanded genetic panels that include *PDYN*. In the present case, the early onset of symptoms and the absence of a familial history are noteworthy, further illustrating the clinical variability already described for SCA23 rather than expanding a previously unknown phenotype. Because parental genetic testing was not performed, a *de novo* origin cannot be confirmed.

This report emphasizes the importance of comprehensive genetic testing in atypical or early-onset ataxia, as well as continued multidisciplinary follow-up and systematic case documentation, to improve understanding of the genotypic and phenotypic variability of SCA23.

## Data Availability

No datasets were generated or analysed during the current study.
